# Bruton tyrosine kinase inhibitor ONO/GS-4059: from bench to bedside

**DOI:** 10.18632/oncotarget.12786

**Published:** 2016-10-20

**Authors:** Jingjing Wu, Mingzhi Zhang, Delong Liu

**Affiliations:** ^1^ Department of Oncology, The first Affiliated Hospital of Zhengzhou University, Zhengzhou, China

**Keywords:** Bruton tyrosine kinase, ONO/GS-4059, ibrutinib

## Abstract

The Bruton tyrosine kinase (BTK) inhibitor, ibrutinib, has been approved for the treatment of chronic lymphocytic leukemia, mantle cell lymphoma, and Waldenstroms macroglobulinemia. Acquired resistance to ibrutinib due to BTK C481S mutation has been reported. Mutations in PLC?2 can also mediate resistance to ibrutinib. Untoward effects due to off-target effects are also disadvantages of ibrutinib. More selective and potent BTK inhibitors (ACP-196, ONO/GS-4059, BGB-3111, CC-292) are being investigated. This review summarized the preclinical research and clinical data of ONO/GS-4059.

## BACKGROUND

A case report in 1952 by Mr. Bruton described a defect with complete absence of gamma globulin synthesis and later the defect was found to be X-linked [1–4]. The gene responsible for the disorder was independently isolated in 1993 by two different groups [5, 6]. The gene, now known as Bruton tyrosine kinase (BTK), is located on the X chromosome and encodes a polypeptide with 659 amino acid residues. BTK protein is now well known to be a downstream molecule of the B cell receptor (BCR) signaling pathway [7–9]. BTK plays a critical role in BCR signaling and B-cell development and function [10–13].

Targeted therapy against biomarker molecules has revolutionized drug development and cancer therapy [14–21]. Novel targeted agents have led to revolutions of lymphoma treatment [22–31]. Since BTK plays a critical role in B cell development and lymphomagenesis, BTK inhibitors have been in active clinical development [32–36]. Early clinical studies have shown that targeting BTK in several B-cell malignancies have been highly effective with tolerable adverse effects [37–43]. The first-generation BTK inhibitor, ibrutinib (PCI-32765), has produced significant clinical response in patients with chronic lymphocytic leukemia (CLL), mantle cell lymphoma (MCL), diffuse large B cell lymphoma (DLBCL) [activated B cell (ABC)-like subtype] as well as in patients with Waldenstrom's macroglobulinemia (WM) [44–46]. Ibrutinib (imbruvica) has been approved for the treatment of CLL, MCL and WM [7, 34, 35, 40, 44–50]. However, there are adverse events like bleeding, rash, atrial fibrillation reported, which were thought to be mostly associated with the off-target effects of ibrutinib [40, 48, 51–58]. Emerging resistance to ibrutinib was also reported [59, 60]. BTK C481S and T316A mutations lead to ibrutinib resistance [61, 62]. Mutations in PLCγ2 can also mediate resistance to ibrutinib. Therefore, more selective and specific BTK inhibitors, such as ACP-196 (acalabrutinib), ONO/GS-4059, BGB-3111, are being explored and developed [20, 33, 34, 36, 61, 63]. In this review we summarized the preclinical research and clinical development of ONO/GS-4059.

## MECHANISM OF ACTIONS AND PROPERTIES OF ONO/GS-4059

ONO/GS-4059 (formerly known as ONO-WG-307) is a highly potent and selective BTK inhibitor [64, 65]. This molecule can inhibit BTK by blocking auto-phosphorylation at the Tyr223 position. Through quantitative phosphoproteomics analysis of phosphorylated BTK proteins by mass spectrometry followed by bio-informatic processing, site-specific phosphorylations were identified [65]. BTK protein was found to be potently and selectively inhibited by ONO/GS-4059 (ONO-WG-307). The IC_50_ of ONO/GS-4059 was in the sub-nanomolar range, which was much lower than the IC_50_ values (above 1 µM) for other tyrosine kinases (Lck, Lyn and Fyn). This suggests that ONO/GS-4059 is highly specific for BTK. In comparison with ibrutinib, ONO/GS-4059 has less bleeding risks (Table 1).

**Table 1:** Comparison of ibrutinib with ONO/GS-4059

	Ibrutinib	ONO/GS-4059
Target	BTK	BTK
off-target effects	++	+*	
AC in trials	Not allowed	allowed	
Platelet inhibition	yes	NA	
Atrial Fibrillation	observed	observed**	
**Approved indications**	CLL/SLL, MCL,WM	none	

Abbreviations: BTK: Bruton tyrosine kinase; AC: anticoagulation; NA: not available /reported; CLL: chronic lymphoid leukemia; SLL: small lymphoid leukemia; MCL: mantle cell lymphoma; WM: Waldenstrom's macroglobulinemia. *: off-target effects were seen in vitro, but overall weaker than those seen with ibrutinib; **: the atrial fibrillation was not thought to be drug- related

ONO/GS-4059 inhibits BTK signaling through AKT and protein kinase D [65, 66]. ONO/GS-4059 exhibits significant activity *in vivo* in the ABC-DLBCL TMD-8 xenograft model*, in vitro* anti-proliferative effects in DLBCL, FL, MCL and CLL cell lines and its combination with other targeted agents [64–68].

## ONO/GS-4059 in preclinical research

ONO/GS-4059 (formerly known as ONO-WG-307) was initially evaluated in cells and in the mouse models [64–68]. In the initial in vitro study, ONO-WG-307 alone and in combination with rituximab were tested in FL and ABC-DLBCL cell lines [64]. The same cells were also used to explore ONO-WG-307 anti-tumor activity in a mouse model. The DLBCL cells were much more sensitive than FL cell lines to single agent OPN-WG-307. In fact, when ONO-WG-307 was combined with rituximab, antagonism of a modest degree was observed in the FL cell lines. Treatment with single agent ONO-WG-307 showed anti-tumor activity in the xenograft models.

The inhibitory effect of ONO/GS-4059 on BTK-dependent signal transduction was further investigated in two tumor cell lines (sensitive and non-sensitive) [65]. The IC_50_ of BTK inhibition in the sensitive cells was 3.59 nmol/L. The inhibition of cellular BTK and ERK phosphorylation were similar in both sensitive and non-sensitive cells. These data demonstrated that the selective inhibition of cell growth by ONO/GS-4059 was due to blocking of BTK-mediated signaling through AKT and cellular protein kinase D.

ONO/GS-4059 was further analyzed *in vivo* for its effects on gene expressions in a xenograft model of the ABC-DLBCL cell line (TMD-8) [66]. ONO/GS-4059 was shown to affect the expression of a core set of genes in a dose-dependent manner. This study confirmed the profound anti-proliferative activity of ONO/GS-4059 by inhibiting BTK in the TMD-8 mouse model.

ONO/GS-4059 was also evaluated in combination with other agents. Combination of idelalisib, a phophotidylinositol 3 kinase (PI3K) inhibitor [69], showed synergistic activity in inhibiting the growth of a subset of DLBCL and MCL cell lines, including 3 ABC-DLBCL cell lines (OCI-LY10, Ri-1, and TMD8) and 2 MCL cell lines (Rec-1 and JMV-2) [67]. Two mechanisms of resistance to BTK inhibitors were identified in the TMD8 cell line: a NF-kB inhibitor A20 mutation (TNFAIP3 Q143*), and a BTK mutation (C481F). TMD8 cells with A20 mutant were sensitive to the combination with ONO/GS-4059 as well as the idelalisib alone. The BTK-C481F mutated TMD8 cells were less sensitive to the idelalisib single agent and addition of ONO/GS-4059 did not enhance the inhibitory activity. In a separate report, TMD8 cells were exposed to high dose idelalisib to establish a resistant cell line [70]. The cell line was resistant not only to idelalisib, but also to both ibrutinib and ONO/GS-4059, confirming that BTK-mediated signaling pathway plays a major role in the B cell survival. These data suggest that combination therapy may be better to overcome resistance in the BTK signaling pathway through the inhibition of PI3 kinase by idelalisib. Quadruple combinations of the B cell receptor pathway inhibitors, entospletinib, ONO/GS-4059, idelalisib, and ABT-199 were studied *in vitro* in primary CLL cells [15, 71, 72]. The study showed that combination treatment synergistically increased the apoptosis in primary CLL cells compared to the individual agents and achieved the maximal levels of apoptosis.

## ONO/GS-4059 in clinical development

The first-in-human phase I study of ONO/GS-4059 was ongoing in relapsed/refractory B-cell malignancies (NCT01659255) [63, 73–75]. In the last update, 90 patients were evaluable for the efficacy and safety. The patients had a spectrum of B cell malignancies (CLL n=28, MCL n=16, DLBCL n=35, FL n=5, WM n=3, MZL n=2 and SLL n=1). The study was safety-driven, dose-escalating in a 3+3 design. The cohorts ranged from 20mg to 600mg once daily with twice-daily regimens of 240mg and 300mg. In the CLL group, 96% (24/25) patients have gained objective response within the first 3 months of therapy. Rapid responses in the lymph nodes were seen in those with concurrent lymphocytosis. High overall response rates were reported in the CLL (96%, 24/25 patients) and in the MCL group (92%, 11/12 patients). Much lower response rate was seen in the patients with non–germinal center DLBCL (35%, 11/31). Therefore, responses of DLBCL were much lower and less durable with most patients dying from disease progression. It was particularly remarkable that those CLL and MCL patients with chromosome 17p deletion and/or TP53 mutation or following allogeneic stem cell transplantation responded rapidly. Rapid absorption and elimination were noted, with a half-life of 6.5 to 8 hours for the BTK inhibitor. ONO/GS-4059 was well tolerated with no maximal tolerated dosage (MTD) reached in the CLL group at the last update. In the lymphoma cohort, 480 mg once daily was the MTD. Most adverse events (AE) were grade 1 or 2. Severe AEs were seen mainly with hematologic toxicities, which were transient and recovered spontaneously [63]. In this trial, anticoagulation was allowed, whereas in ibrutinib trials, anticoagulation was not. Increased bleeding was not observed in this report in the 28 patients who were on anticoagulation. From the early phase studies, the BTK inhibitor ONO/GS-4059 appears to have a favorable safety profile in patients with relapsed/refractory B-cell malignancies. Its high response rates in poor risk patients with CLL and MCL are particularly remarkable.

## CONCLUSION AND FUTURE DIRECTIONS

ONO/GS-4059 is a novel, potent and selective second-generation inhibitor of BTK. It can inhibit auto-phosphorylation of the BTK at the Tyr223 position through the ERK, AKT and PKD signaling pathways. This drug exhibits significant antitumor activity in pre-clinical models and in phase I clinical trials. To date, the efficacy and tolerability of ibrutinib have led the way to further development of novel agents in the treatment of B cell malignancies. New generation BTK inhibitors have improved selectivity and efficacy, however, they are still in early stage of clinical trials. In addition, acquired resistance to the BTK inhibitors have been reported. With the rapid clinical development of novel agents of bispecific antibodies [76–78], antibody-drug conjugates [79, 80], immune checkpoint blockers [81–83], and CAR-T for cancer immunotherapies [84–87], combinations of BTK inhibitors with novel agents may overcome acquired resistance in refractory B cell malignancies [88].

## Abbreviations

BTK: Bruton tyrosine kinase
ORR: overall response rate
PR: partial response
SD: stable disease
DLT: dose-limiting toxicity
MTD: maximal tolerated dose.
